# Severe Hypoxemia Caused by High-Output Heart Failure and Pulmonary Arteriovenous Malformations in Hereditary Hemorrhagic Telangiectasia

**DOI:** 10.1016/j.jaccas.2021.10.008

**Published:** 2021-12-01

**Authors:** Hiroaki Yamamoto, Chieko Itamoto, Tomomi Yamaguchi, Tomoki Koshyo

**Affiliations:** a Department of Cardiology, Nagano Chuo Hospital, Nagano, Japan; b Department of Genetic Medicine, Shinshu University School of Medicine, Matsumoto, Japan; c Center for Medical Genetics; and; d Division of Clinical Sequencing, Shinshu University School of Medicine, Matsumoto, Japan; e Research Center for Supports to Advanced Science, Shinshu University, Matsumoto, Japan

**Keywords:** chronic heart failure, genetic disorders, genotype, phenotype, right-sided catheterization, AVM, arteriovenous malformation, CT, computed tomography, HAVM, hepatic arteriovenous malformation, HHT, hereditary hemorrhagic telangiectasia, HF, heart failure, PAVM, pulmonary arteriovenous malformation

## Abstract

A man affected by hereditary hemorrhagic telangiectasia who had chronic severe hypoxemia is presented. This hypoxemia was synergistically caused by high-output heart failure due to severe hepatic shunts and multiple pulmonary arteriovenous shunts. The symptomatic combination is rare, and genetic testing showed a novel endoglin mutation. (**Level of Difficulty: Advanced.**)

## Central Illustration

**Figure:**
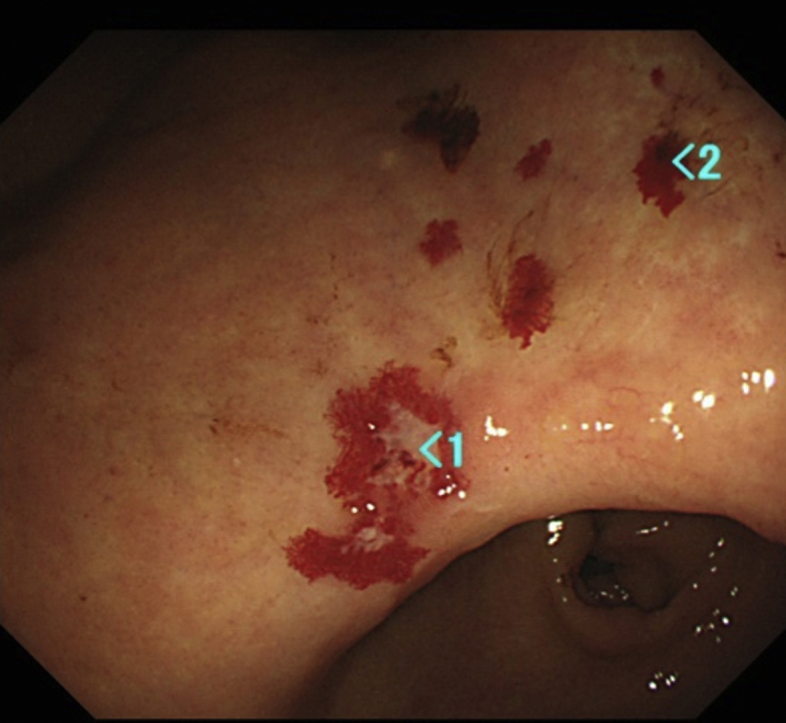

Hereditary hemorrhagic telangiectasia (HHT) is an autosomal dominant genetic disorder characterized by epistaxis, mucocutaneous telangiectasia, and arteriovenous malformations (AVMs). HHT is one of the most common diseases that causes high-output heart failure (HF), which is defined as fulfillment of the Framingham criteria and demonstration of a cardiac index over 4 L/min per m^2^ (1). Interestingly, this HF is caused not by a heart disorder but by a liver disorder. Representative disease-causing genes are *ENG*, which encodes endoglin (HHT1), and *ACVRL-1*, which encodes ALK-1 (HHT2). The major phenotypes are pulmonary arteriovenous malformation (PAVM) and hepatic AVM (HAVM). These phenotypes are thought to partly depend on genotypes that include mutant genes. Patients with HHT1 frequently have PAVMs, but HAVMs are rare complications in male patients (2). By contrast, HHT2 patients more commonly have HAVMs but seldom have PAVMs.

**Learning Objectives**

• To recognize that hepatic AVMs in HHT cause high-output heart HF.
• To know that different mutated genes show different organ-AVMs in HHT patients.

## History of Presentation

Here we report the case of a patient with an endoglin gene mutation that was not included previously in the HHT mutation database (3,4). This patient had large PAVMs that caused hypoxemia resulting from a right-to-left shunt as well as HAVMs that showed high-output HF. A severe symptomatic combination of PAVMs and HAVMs that synergistically cause severe hypoxemia is rare.

## Medical History

**Figure 1:**
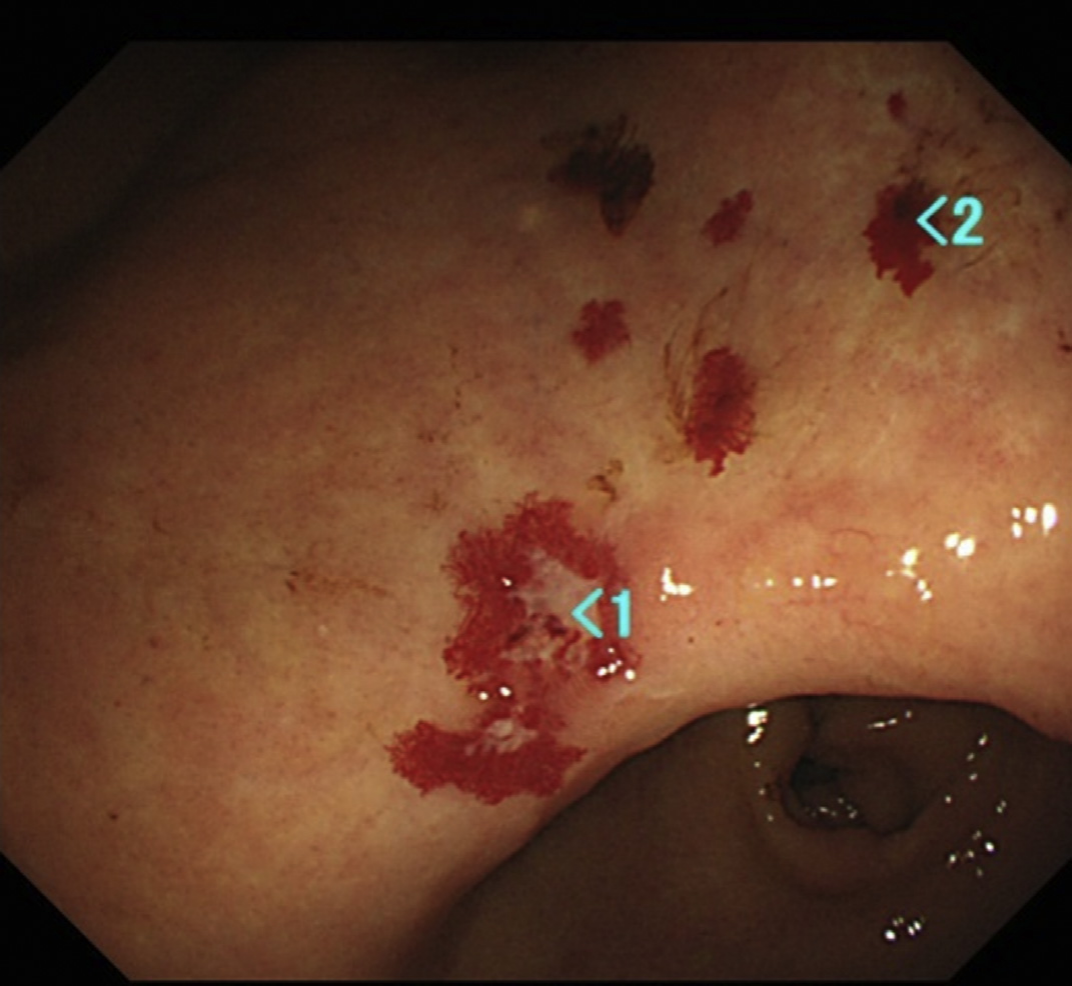
Telangiectasia in the Stomach Multiple telangiectasia in the stomach were observed.

An 87-year-old man was admitted to our hospital with pretibial edema and aggravated dyspnea. At age 77, he had experienced upper gastrointestinal bleeding caused by mucous telangiectasia (Figure 1). At age 86, he had experienced moderate dyspnea with low oxygen saturation of nearly 80%. The symptoms progressively worsened, and he was admitted to our hospital.

## Differential Diagnosis

**Figure 2:**
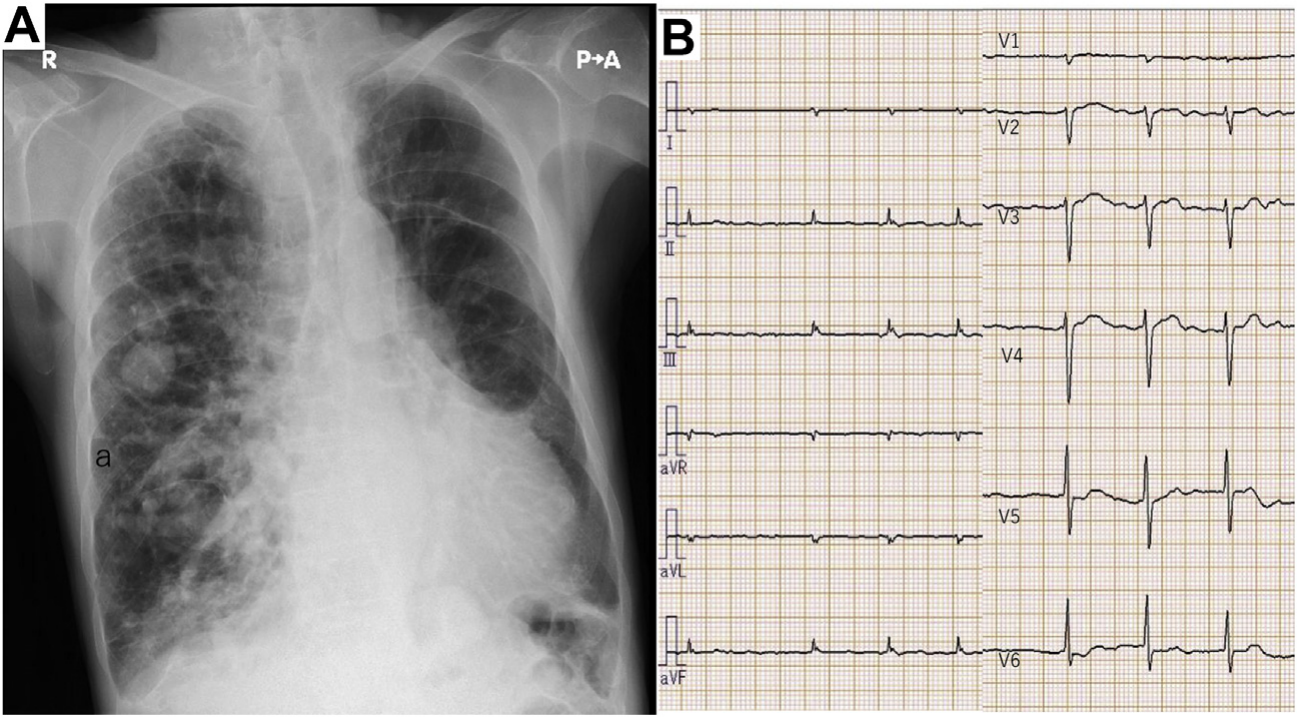
Chest X-Ray and Electrocardiogram on Admission **(A)** Chest X ray showed cardiomegaly and lung congestion with multiple coin lesions. **(B)** Electrocardiogram showed atrial fibrillation.

**Figure 3:**
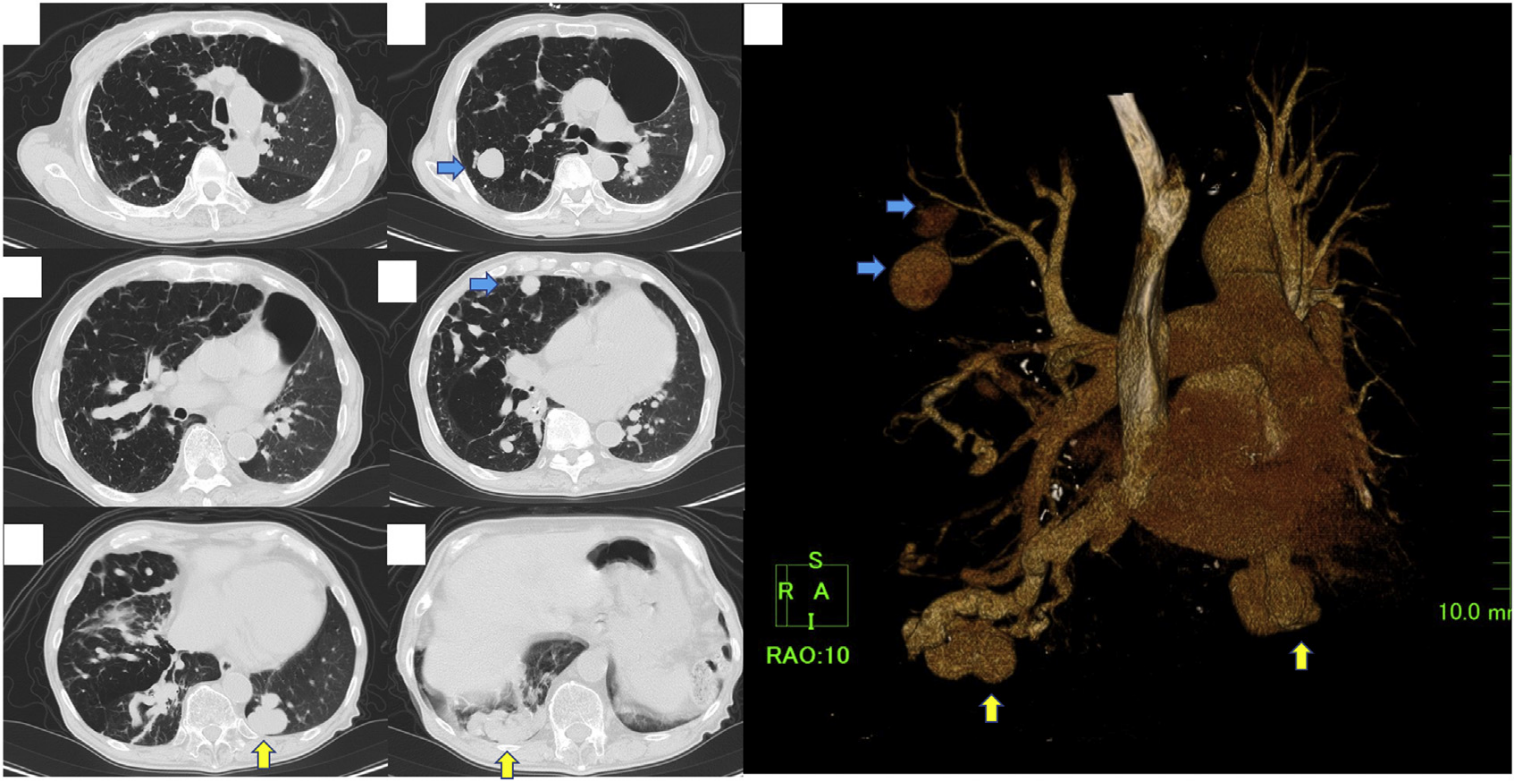
Computed Tomography of the Lung Computed tomography revealed multilobular fistulas **(yellow arrows)** and aneurysms **(blue arrows)**.

Laboratory data showed arterial oxygen tension of 55.6 mm Hg, and a brain natriuretic peptide level of 508.6 ng/mL (Table 1). A chest X-ray revealed multiple coin lesions (Figure 2). Computed tomography (CT) revealed four PAVMs, 2 of which were multilobular arteriovenous fistulas and 2 of which were aneurysms (Figure 3).

**Table 1:** Laboratory Data Including Echocardiographic Data

Blood gas analysis (room air)	
PH	7.464
PCO_2_	49.7 mm Hg
PO_2_	55.6 mm Hg
HCO_3-_	35.2 mEq/L
sO2	89.4 %
Blood and blood chemistry	
WBC	4,850 /μL
RBC	461×10^4^/μL
Hemoglobin	14.6 g/dL
Hematocrit	47.4 %
Platelet count	11.8×10^4^/μL
Total protein	5.5 g/dL
Albumin	3.8 g/dL
AST	35 IU/L
ALT	49 IU/L
LDH	299 IU/L
CK	68 IU/L
Total bilirubin	0.7 mg/dL
BUN	35.6 mg/dL
Creatinine	0.97 mg/dL
Uric acid	8.7 mg/dL
Sodium	140 mEq/L
Potassium	4.6 mEq/L
Plasma glucose	110 mg/dL
Ferritin	13 ng/mL
BNP	508.6 ng/mL
Fibrinogen	209.7 mg/mL
FDP	2.6 μg/mL
D-dimer	1.0 μg/mL
Echocardiogram	
IVST	8 mm
LVPWT	10 mm
LVDd	49 mm
LVDs	26 mm
LVEF	79 %
E wave amplitude	177 cm/s
DT	268 ms
Cardiac output	6.5 L/min
Cardiac index	4.17 L/min/m^2^
Tricuspid regurgitation	grade 3

ALT = alanine aminotransferase; AST = aspartate transaminase; BNP = brain natriuretic peptide; BUN = blood urea nitrogen; CK = creatine kinase; DT = deceleration time; E/A = early diastolic filling velocity/atrial filling velocity; FDP = fibrin/fibrinogen degradation products; HbA1c = hemoglobin A1c; IVST = interventricular septum thickness; LDH = lactate dehydrogenase; LVDD, left ventricular diastolic dimension; LVEF = left ventricular ejection fraction; LVPWT = left ventricular posterior wall thickness; LVSD = left ventricular systolic dimension; RBC = red blood cell count; WBC = white blood cell count.

**Figure 4:**
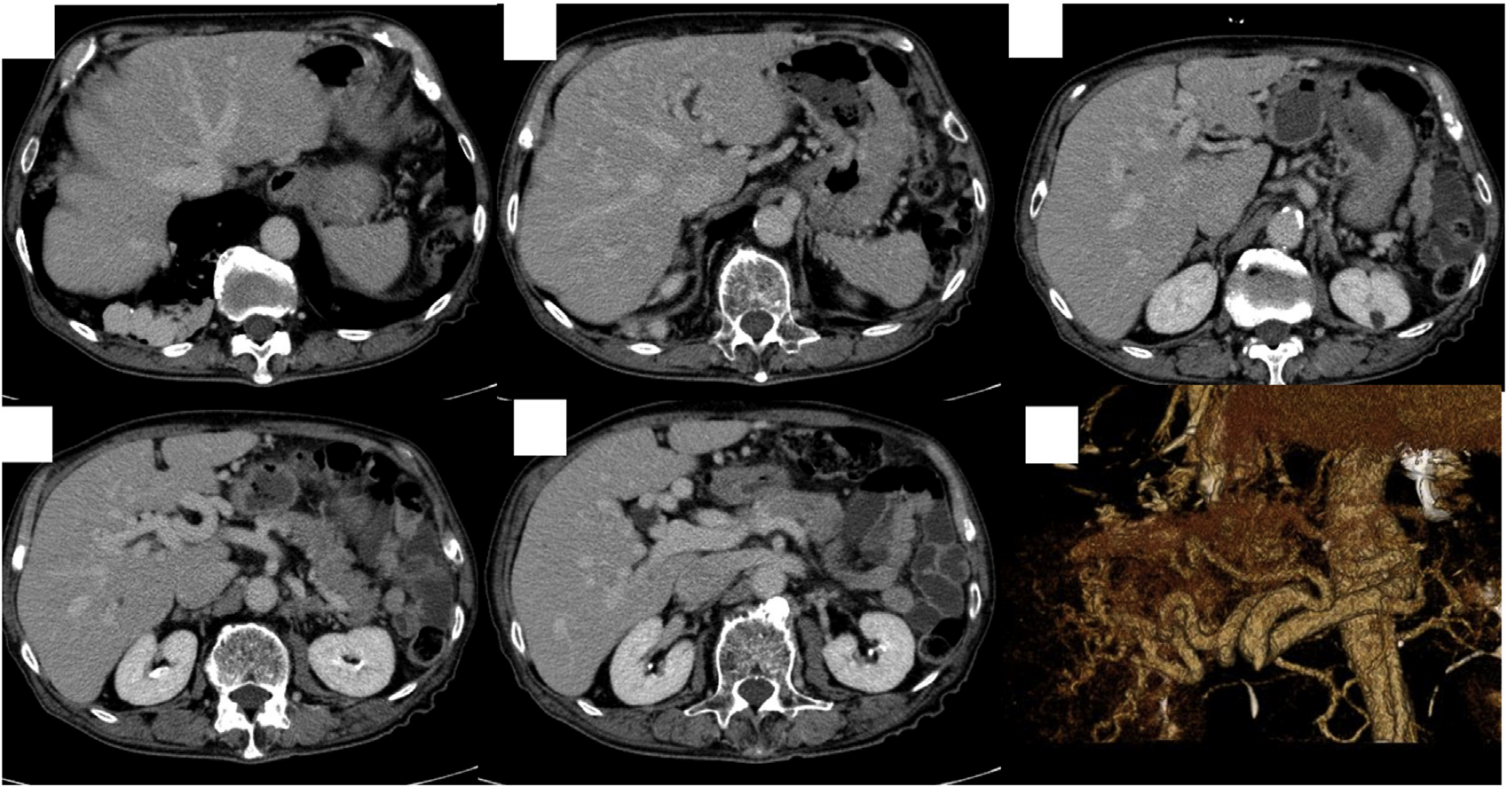
Computed Tomography of the Liver Computed tomography showed multiple arteriovenous shunts.

**Figure 5:**
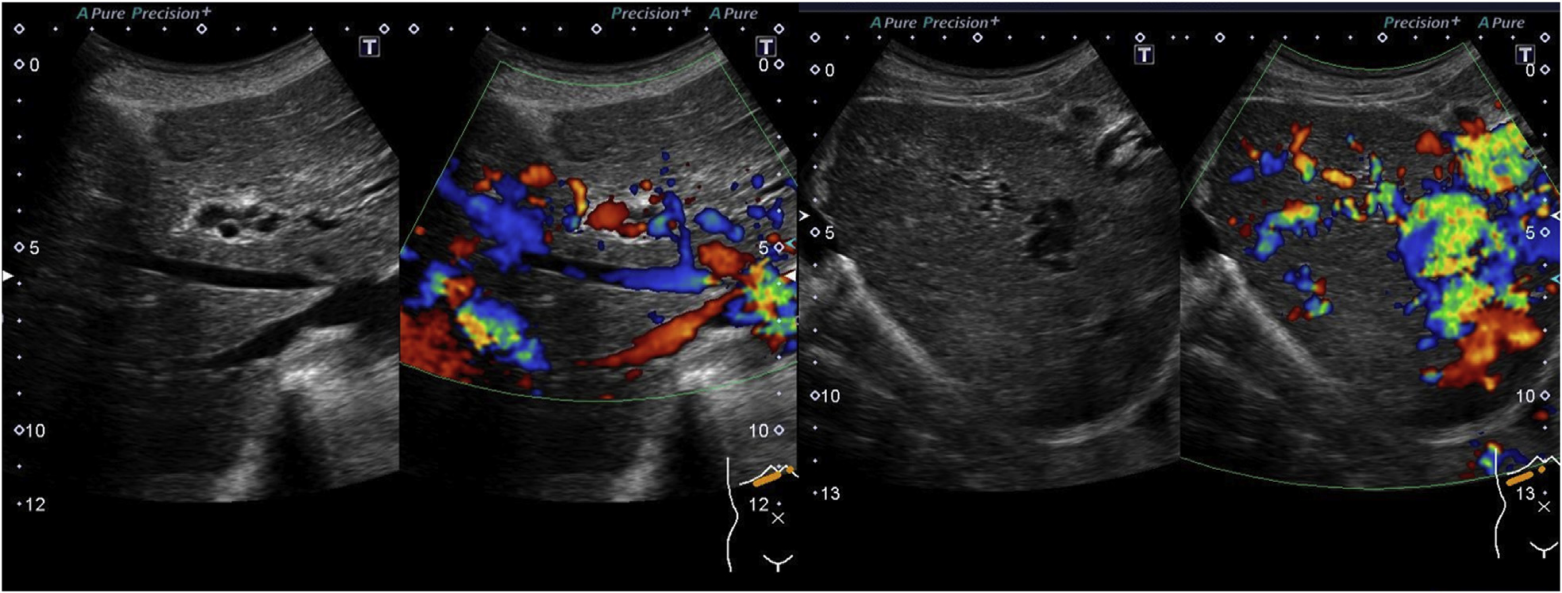
Ultrasound of the Liver Ultrasound represented abundant intrahepatic vessels.

CT angiograms also revealed HAVMs, arteriovenous shunts, and port-venous shunts (Figure 4). An ultrasound showed abundant intrahepatic vessels (Figure 5). Echocardiography showed that the patient had a high cardiac output of 6.50 L/min (Video 1). His daughter was also found to have PAVMs and HAVMs. Thus, this patient received a definite diagnosis of HHT, fulfilling all 3 Curaçao diagnostic criteria (5).

## Management

We performed cardiac catheterization. The hemodynamic data showed a cardiac output of 6.87 L/min and pulmonary arterial wedge pressure of 20 mm Hg (Table 2). A pulmonary angiogram showed 4 large AVMs. This, combined with the pretibial edema and lung congestion, led to a diagnosis of high-output HF. To ameliorate the hypoxemia, we performed coil embolization of the largest left lobe fistula with the following procedure. First, a guidewire was inserted into the PAVM. Next, the flow was stopped with an occlusion balloon that was attached to the guiding catheter; 3 IDC coils were inserted at the entry of the aneurysm to make the cage. Then, 4 interlocking coils were packed. During this procedure, the PAVM was successfully isolated, and the fistula was completely occluded (Videos 2, 3, and 4). After treatment, oxygen saturation increased from 85% to 90%. The patient was discharged a few days later. About 2 months later, he was admitted again because of disturbances in his consciousness and the appearance of flapping tremors. His serum ammonia concentration was 237 mg/dL (normal range 12-66 mg/dL). We started branched amino acid therapy, which gradually ameliorated the patient’s condition.

**Table 2:** Right Heart Catheterization Data

Blood pressure	120/33 mm Hg
Heart rate	72 beats/min
Right atrial pressure (mean)	10 mm Hg
Pulmonary arterial pressure	38/20 mm Hg
Pulmonary arterial pressure (mean)	24 mm Hg
Pulmonary arterial wedge pressure	20 mm Hg
Cardiac output (thermodilution)	6.87 L/min
Cardiac index	4.41 L/min per m^2^
Systemic vascular resistance	605 dynes · s/cm^5^
Pulmonary arterial resistance	46 dynes · s/cm^5^

**Figure 6:**
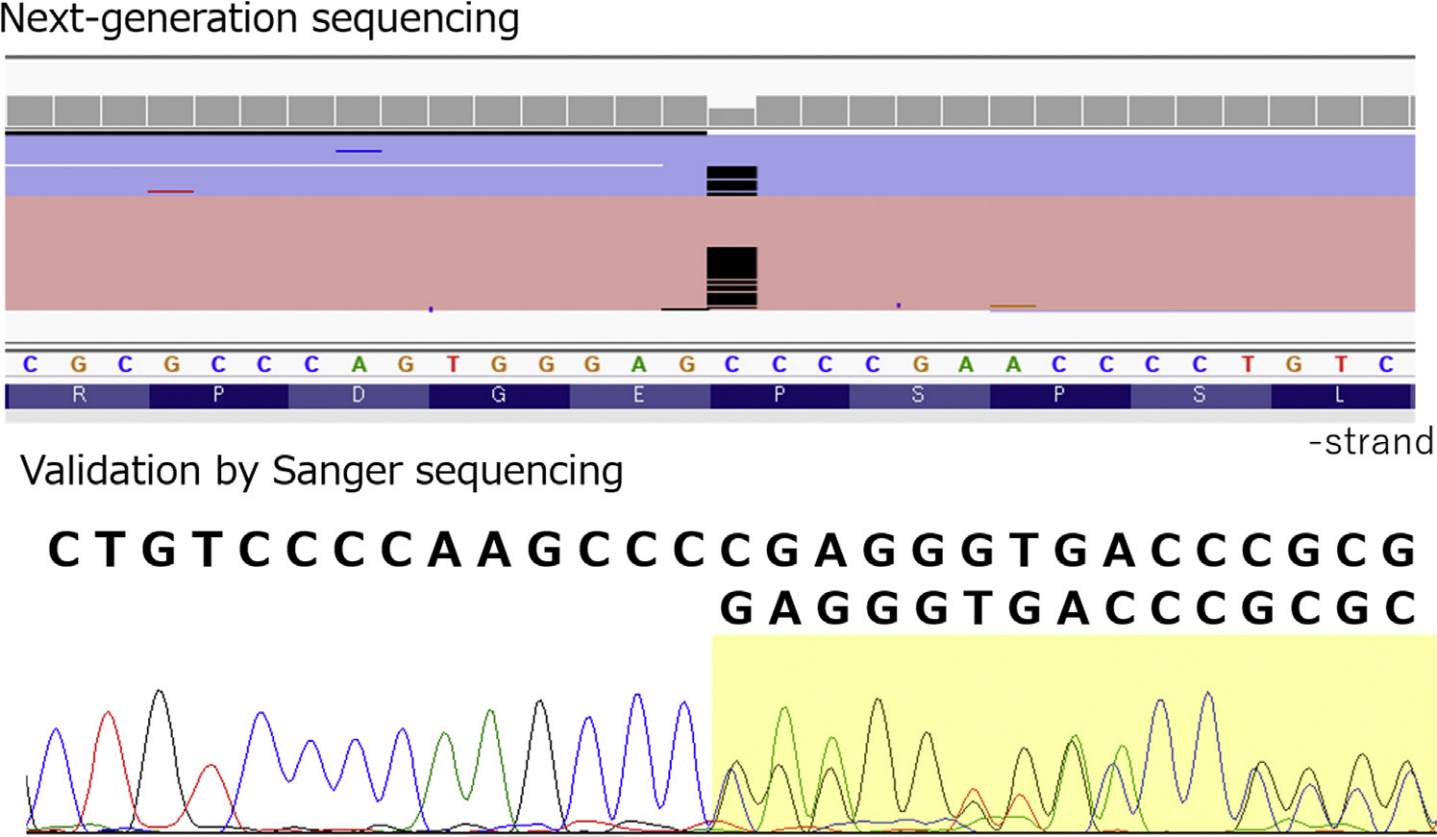
Genetic Testing Revealed an *ENG* 1-Base Deletion A heterozygous frameshift variant in *ENG* (NM_000118.3:c.1572del) was detected.

A male patient with both severe symptomatic PAVMs and HAVMs is extremely rare. Therefore, we conducted custom panel–based next-generation sequencing for hereditary connective tissue disorders including 55 relevant genes. A heterozygous frameshift variant in *ENG* (NM_000118.3:c.1572del: p.Glu525Argfs∗27) was detected both in the patient and in his affected daughter, who has PAVMs and HAVMs (Figure 6). The variant was not registered in the HHT mutation database (3), in ClinVar (4), or in several population databases. We concluded that the variant was pathogenic according to the ACMG/AMP guideline of 2015. It was suggested that the frameshift at exon 12 did not produce normal endoglin proteins but instead created truncated abnormal endoglin proteins.

## Discussion

HHT is diagnosed with the Curaçao criteria, which are composed of 4 criteria: recurrent epistaxis, mucocutaneous telangiectasias, multiple AVMs, and family history. If the patient fulfills more than 3 of the 4 criteria, then the diagnosis becomes definite (5).

PAVMs induce problems such as hypoxemia and cerebral embolism. Whereas HAVMs also induce various problems, the first problem caused by a HAVM is high-output HF due to an AV shunt. This is one of the most frequent causes of high-output HF observed in white patients (1). The second problem is hepatic encephalopathy, which is induced by port-venous shunts. The other problems are portal hypertension and biliary disease. All of them are life-threatening disorders, which is why the second international guideline of HHT (6) stresses the importance of HAVMs.

The patient presented here had a symptomatic combination of PAVMs and HAVMs that collectively caused severe hypoxemia. Many reports mentioned that patients who have an *Endoglin* mutation (HHT1) tend to have PAVMs. However, in these patients, HAVMs are very rare. By contrast, patients who have an *ACVRL1* mutation (HHT2) have HAVMs more frequently, and PAVMs are rare. There are many reports concerning genotype-phenotype correlations of HHT in which the gender difference is important (2,7). Letteboer et al (2) reported that in HHT1, only 1 of 56 male patients had HAVMs, whereas 9 of 88 female patients had HAVMs. Kilian et al (7) reported that only 1 of 101 male children with HHT1 had HAVMs. Interestingly, Dupuis-Girod et al (8) published an article that demonstrated the effectiveness of bevacizumab in the treatment of high-output HF due to HAVM. Of 25 patients recruited for this treatment, 24 patients (96%) were female, and 22 patients (88%) were HHT2 patients. Thus, patients who show high-output HF are usually female HHT2 individuals.

According to these statistics, our male HHT1 patient showed a very unique phenotype, with multiple PAVMs and 2 types of symptomatic HAVMs. Whether these phenotypes came from the newly discovered *ENG* variant is uncertain. Considering that the daughter has the same phenotype and genotype, this newly discovered variant may be involved.

From a clinical perspective, this combination generated severe hypoxemia caused the intrapulmonary shunt and high-output HF. To treat these complex conditions, the most reliable and evidence-rich procedure is coil embolization of the PAVM. The other treatments are procedures that are directed at treating HAVMs, such as liver transplantation and bevacizumab treatment. Coil embolization to HAVMs was not recommended by the second international guideline (6). Dupuis-Girod et al (8) demonstrated that bevacizumab lowered cardiac output significantly and improved symptoms. In Japan, this drug is not yet approved for the treatment of HHT. Liver transplantation is an established treatment for HHT patients with severe HAVMs (6).

## Follow-Up

The patient’s condition became stable, and he was discharged after 1 month of treatment.

## Conclusions

Prominent PAVMs and HAVMS in an HHT1 male patient caused hypoxemia and high-output HF, and novel genetic mutations that cause truncated endoglin protein were discovered in his family.

## Funding Support and Author Disclosures

The authors have reported that they have no relationships relevant to the contents of this paper to disclose.

## Appendix

Supplemental Video 1 Echocardiographic View Showing High-Output State

Supplemental Video 2 Pulmonary Angiogram Showing Pulmonary Aneurysms

Supplemental Video 3 Pulmonary Angiogram Showing Arteriovenous Fistulas

Supplemental Video 4 Arteriovenous Fistula After Coil Embolization
